# NFAT1-regulated IL6 signalling contributes to aggressive phenotypes of glioma

**DOI:** 10.1186/s12964-017-0210-1

**Published:** 2017-12-19

**Authors:** Yang Jiang, Sheng Han, Wen Cheng, Zixun Wang, Anhua Wu

**Affiliations:** Department of Neurosurgery, The First Hospital of China Medical University, Nanjing Street 155, Heping District, Shenyang, 110001 China

**Keywords:** Glioma, Glioblastoma, IL6, IL6R, NFAT1

## Abstract

**Background:**

We previously demonstrated that the local immune status correlated with the glioma prognosis. Interleukin-6 (IL6) was identified as an important local immune-related risk marker related to unfavourable prognosis. In this study, we further investigated the role and regulation of IL6 signalling in glioma.

**Methods:**

The expression and prognostic value of IL6 and the IL6 receptor (IL6R) were explored in The Cancer Genome Atlas (TCGA) and REMBRANDT databases and clinical samples. Functional effects of genetic knockdown and overexpression of IL6R or IL6 stimulation were examined in vitro and in tumours in vivo. The effects of the nuclear factor of activated T cells-1 (NFAT1) on the promoter activities of IL6R and IL6 were also examined.

**Results:**

High IL6- and IL6R-expression were significantly associated with mesenchymal subtype and IDH-wildtype gliomas, and were predictors of poor survival. Knockdown of IL6R decreased cell proliferation, invasion and neurosphere formation in vitro, and inhibited tumorigenesis in vivo. IL6R overexpression or IL6 stimulation enhanced the invasion and growth of glioma cells. TCGA database searching revealed that IL6- and IL6R-expression were correlated with that of NFAT1. In glioma cells, NFAT1 enhanced the promoter activities of IL6R and IL6, and upregulated the expression of both IL6R and IL6.

**Conclusion:**

NFAT1-regulated IL6 signalling contributes to aggressive phenotypes of gliomas, emphasizing the role of immunomodulatory factors in glioma malignant progression.

**Electronic supplementary material:**

The online version of this article (10.1186/s12964-017-0210-1) contains supplementary material, which is available to authorized users.

## Background

Glioma is the most common primary brain tumour in adults, and the clinical outcome of gliomas remains unsatisfactory after current standard treatments [1]. New treatment strategies are therefore urgently needed, and immunotherapy is regarded as one of the most promising ways to cure gliomas [2]. However, the immune-related biological and molecular characteristics within the glioma microenvironment that significantly influences the efficacy of immunotherapy need to be further clarified.

Local and systemic immune disorders contribute to the development and progression of gliomas [3]. Using the Chinese Glioma Genome Atlas (CGGA) and The Cancer Genome Atlas (TCGA) database, we previously established eight immune-related genes as local immune signatures for glioblastoma (GBM) that could independently identify patients with a high risk of reduced survival. Interleukin-6 (IL6) was one of the eight immune-related genes with the greatest prognostic value in GBM [4].

IL6 is a pleiotropic cytokine that was first recognized for its ability to promote the population expansion and activation of T cells, the differentiation of B cells, and the regulation of the acute-phase response [5, 6]. Classically, IL6 binds to its specific receptor (IL6R) leading to the dimerization of the signal transducer receptor (IL6ST), and subsequently activates the JAK/STAT pathway [6]. Recent studies demonstrated aberrant IL6 production and secretion in a large variety of malignant tumours, including breast cancer [7], ovarian cancer [8], lung cancer [9] and GBM [10–15], revealing the oncogenic effects of IL6 signalling. In gliomas, upregulation of IL6 expression correlates with poor patient survival [13, 14], while ablation of IL-6 prevents glioma formation in a mouse model [16]. Moreover, IL6 generates an inflammatory microenvironment and promotes glioma stem-like cells (GSCs) survival and growth [11, 17]. Nevertheless, previous studies mainly focused on IL6 and the research regarding IL6R remains limited. Moreover, the regulatory factors of IL6 signalling are largely unknown. Therefore, in the present study, we examined the role of IL6 and IL6R along with their regulatory factors in gliomas.

## Methods

### Cell culture and cell treatment

Human glioma cell line T98G was purchased from American Type Culture Collection (ATCC, Manassas, VA, USA) in December 2016. U87 and U251 cells were purchased from the Chinese Academy of Sciences cell bank (Shanghai, China) in January and September 2016, respectively. LN229 and SNB19 cells were obtained as a gift from Professor Tao Jiang, Department of Molecular Neuropathology, Beijing Neurosurgical Institute. Cells were maintained in Dulbecco’s modified Eagle’s medium (DMEM, HyClone, Logan, UT, USA), supplemented with 10% foetal bovine serum (FBS, Gibco, Carlsbad, CA, USA) and 1% penicillin/streptomycin (Gibco) at 37°C with 5% CO_2_.

Patient-derived primary glioma cells (P1, P2, N1, N2, C1, C2, M1, and M2) were established as previously described [18]. The expression of mRNA markers (EGFR, FN1, YKL40, NEFL, PDGFRA and OLIG2) was examined using real-time PCR (Additional file 1: Figure S1) for the molecular classification of the primary glioma cells [19]. Neurosphere culture was performed and the expression of the stem cell marker, CD133, was examined by immunofluorescence using anti-CD133 (1:100; ab19898, Abcam, Cambridge, UK). The multi-lineage differentiation capacity of GSCs was examined using anti-glial fibrillary acidic protein (GFAP, ab7260, Abcam) and anti-β III tubulin (ab78078, Abcam).

Recombinant human IL-6 (R&D Systems, Minneapolis, MN, USA) was dissolved in sterile PBS (HyClone) at 100 μg/ml according to the manufacturer’s instructions, followed by different concentration preparations for glioma cell treatment.

### Real-time PCR

Mini-BEST Universal RNA Extraction kit (TaKaRa, Kyoto, Japan) was used to isolate total RNA according to manufacturer’s instructions. Prime-Script RT Master Mix (TaKaRa) was used to synthesis first-strand cDNA, followed by qPCR (PCR LightCycler480, Roche Diagnostics Ltd., Basel, Switzerland) detection using SYBR Green Master Mix (TaKaRa). Each sample was run four times and β-actin was used as the internal control. The PCR primers are shown in Additional file 2: Table S1.

### Western blotting analysis

Western blotting analysis was performed as described previously [20]. Briefly, a total cell protein extraction kit (KeyGen Biotechnology, Nanjing, China) was used to extract total protein. An equivalent amount of protein from each sample was electrophoresed and transferred to a nitrocellulose membrane and blocked with 2% bovine serum albumin (BSA). The membranes were then incubated with anti-IL6 (1:2000, ab6672, Abxam), IL6R (1:1000, ab128008, Abcam) or NFAT1 (1:1000, ab2722, Abcam) at 4°C overnight. Membranes were washed four times with TBST and incubated with the appropriate secondary antibody. Bands were detected using a chemiluminescence kit (Beyotime Biotechnology, Beijing, China) and quantified with Image J (National Institutes of Health, Bethesda, MD, USA).

### Lentiviral vector construction and transfection

Short hairpin (sh)RNA-mediated knockdown of IL6R and the nuclear factor of activated T cells-1 (NFAT1) were performed as previously described [20]. Plasmids encoding shRNAs targeting IL6R, NFAT1 or control plasmids were obtained from Gene-Chem (Shanghai, China). Lentivirus-based vector was constructed for IL6R and NFAT1 overexpression (Gene-Chem). The transfected cells were screened with 10 μg/ml puromycin (Sigma, Santa Clara, CA, USA) for 15 days and the effectiveness of gene silencing and overexpression were assessed using western blotting.

### Cell viability assay

Cell viability was measured using a CellTiter 96® AQueous Non-Radioactive cell proliferation assay kit (Promega, Madison, WI, USA) according to the manufacturer’s instructions. Briefly, cells were cultured in 96-well plates at a density of 1 × 10^3^ cells/well for 24, 48, 72, 96 and 120 h. In some cultures, cells were treated with IL6 at 10, 20,and 100 ng/ml, respectively, after which 20 μl of MTS was added into each well, followed by 3 h incubation at 37°C. An ultraviolet spectrophotometer (Thermo Fisher Scientific, Waltham, MA, USA) was used to detect the absorbance at 495 nm.

### Transwell invasion assay

The transwell invasion assay was performed as previously described [21]. Cells were allowed to invade the matrigel-coated filters toward the lower compartment for 20 h. In some experiments, cells were treated with 20 ng/ml of IL6. Invasive cells were counted and photographed using a microscope (Olympus, Tokyo, Japan).

### Wound healing assay

Cells were seeded in six-well plates, grown to a 100% confluence and a 1 ml pipette tip was used to scratch a neat and straight line in each well. Each well was washed with PBS twice to remove debris, and fresh serum-free DMEM was added. In some cultures, cells were treated with 20 ng/ml of IL6. Five fields of each wound were monitored at 0 and 24 h to evaluate the migration of cells. The wound healing rate was calculated with Image J (National Institutes of Health).

### Colony formation assay

Cells were seeded in six-well plates at a density of 300 cells/well and cultured in fresh DMEM with 10% FBS for 15 days. In some cultures, cells were treated with 20 ng/ml of IL6. A 1% crystal violet solution (Solarbio Science & Technology, Beijing, China) was used to stain the colonies. Colonies with a diameter larger than 20 μm were counted under a microscope (Olympus) and the colony formation rate was calculated.

### TUNEL assay

TUNEL assay was performed to detect the apoptotic cells after 3 days in culture using the TdT-FragEL DNA Fragmentation Detection kit (QIA33; Merck, Darmstadt, Germany) according to the manufacturer’s instructions. TUNEL-positive cells were counted and the apoptotic rate was calculated as follows: positive cells / (positive cells + negative cells) × 100%.

### Neurosphere formation assay

The neurosphere formation assay was used to assess the self-renewal capacity of GSCs as previously described [18]. In brief, cells were dissociated from neurospheres, seeded in 24-well plates at a density of 200 cells/well, and cultured for 7 days. In some experiments, cells were treated with 20 ng/ml IL6.

### Xenografts

Six-week-old female BALB/c nude mice were purchased from Beijing Vital River Laboratory Animal Technology Co., Ltd. and bred in laminar flow cabinets under specific pathogen free conditions in the Laboratory Animal Center of China Medical University.

M1 or N2 cells were implanted intracranially in anaesthetized nude mice using a stereotaxic apparatus as previously described [20]. We observed mice daily for signs of distress or death and tumour growth was assessed. For subcutaneous tumour models, cells were harvested at a density of 1 × 10^7^ cells/ml and injected (250 μl) into the back flanks of anaesthetized mice. The mice were kept feeding for 5 weeks and the tumour size was measured every 5 days with callipers (Precision Instruments Co., Shanghai, China). To calculate the tumour volume, this formula was used: V = (D × d^2^) / 2, where D represents the longest diameter and d represents the shortest diameter. The mice were sacrificed by cervical spine dislocation when they exhibited signs of distress and each tumour was weighed.

### Immunohistochemistry (IHC)

The expression of IL6R was evaluated using IHC in tumour specimens as previously described [20]. Paraffin-embedded sections were labelled with primary antibody against IL6R (1:200, ab128008, Abcam) and samples were imaged under a BX-51 light microscope (Olympus).

### Enzyme-linked immunosorbent (ELISA)

ELISA was performed as previously described [22]. An ELISA kit for IL6 was obtained from R&D Systems.

### Immunofluorescence

Immunofluorescence staining was carried out as previously described [20, 22]. Antibodies (1:100) against IL6R (ab128008, Abcam) and NFAT1 (ab2722, Abcam) were used to detect the co-expression of these two proteins.

### Luciferase activity analysis

IL6 and IL6R promoters were cloned from U87 cells to encompass 1923 (IL6) or 446 (IL6R) base pairs upstream of the respective transcriptional initiation sites. QuikChange II XL Site-Directed Mutagenesis Kit (Agilent Technologies, Santa Clara, CA, USA) was used for direct site mutagenesis of NFAT1 binding sites following the manufacturer’s instructions. After 48 h, the cells were lysed and luciferase activity was examined using the Dual Luciferase Reporter Assay System (Promega), according to the manufacturer’s instructions.

### Chromatin immunoprecipitation (ChIP) assays

ChIP assays were performed using EZ-ChIP ™ Immunoprecipitation Kit (Millipore, Billerica, MA, USA) according to the manufacturer’s instructions. The chromatin complexes were immuno-precipitated with anti-NFAT1 antibody (1:100, ab2722, Abcam) and the purified DNA samples were analysed with qPCR using primer pairs for the NFAT1 binding site in the IL6 promoter f: 5**′**-CTTCCCACAGTTTGCCCTTTC-3**′** and r: 5**′**-AGTAGGAGCAAGACGCAAGC-3**′**; IL6R promoter f: 5**′**-TGCCCGTTCTTGGTTT-3**′** and r: 5**′**-TGTTCCTGTCTGTGGGCA-3**′**. All reactions were performed in triplicate.

### Statistical analysis

All experiments were repeated at least three times and results were presented as mean ± SEM. The *chi*-square test, *t*-test and analysis of variance were used to evaluate the statistical significance among groups. The TCGA glioma dataset and National Cancer Institute’s Repository for Molecular Brain Neoplasia Data (REMBRANDT) database were explored as previously described [4]. In brief, TCGA and REMBRANDT data were accessed and processed via the GlioVis online platform [23–25] (http://gliovis.bioinfo.cnio.es/). The raw. CEL files of Affymetrix expression arrays were analysed using the Bioconductor suite in R. For robust multi-array average normalization followed by quantile normalization, the “affy” package was used. For RNA-sequencing data, the normalized count reads from the pre-processed data were log2 transformed after adding a 0.5 pseudo-count. More details of the standardized normalization, packages and statistical calculations can be found on the GlioVis. Differences in survival rates were analysed with the log-rank test and Kaplan–Meier analysis. The Pearson correlation analysis was used to analyse the relation between the levels of NFAT1 and the levels of IL6 and IL6R, respectively. Two-tailed *P*-values <0.05 were considered statistically significant. SPSS v.19.0 (SPSS Inc., Chicago, IL, USA) software was used for statistical analyses.

## Results

### High expression of IL6 and IL6R is associated with aggressive subtypes of gliomas

**Fig. 1:**
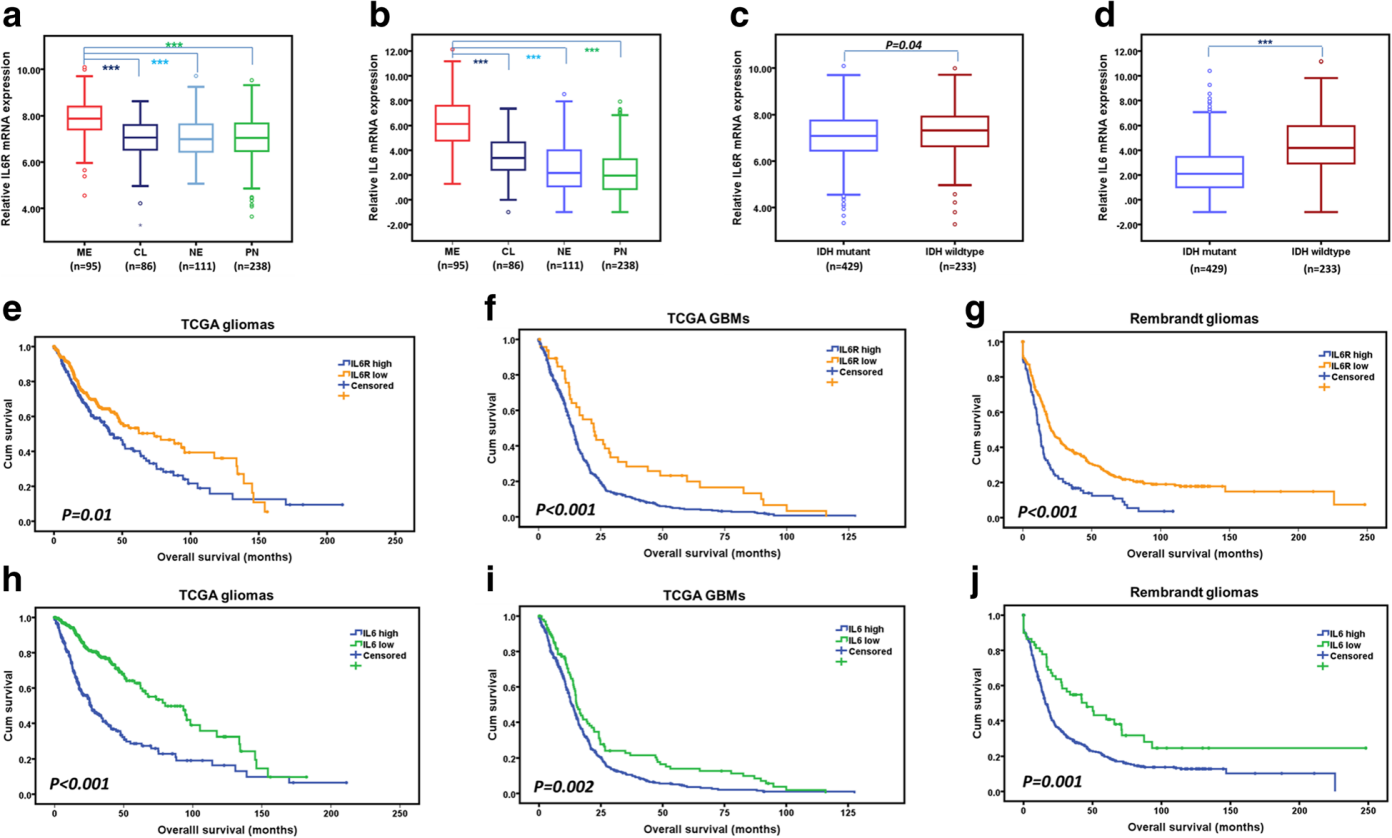
Associations between IL6/IL6R expression and the clinic-pathologic features of gliomas. **a**–**b** The expression of IL6R (**a**) and IL6 (**b**) were shown according to molecular subtypes of TCGA gliomas. **c**–**d** The expression of IL6R (**c**) and IL6 (**d**) were shown according to IDH status of TCGA gliomas. **e**–**f** Prognostic significance of IL6R in TCGA gliomas (**e**) and GBMs (**f**). **g** The prognostic significance of IL6R was validated in REMBRANDT gliomas. **h–i** Prognostic significance of IL6 in TCGA gliomas (**h**) and GBMs (**i**). **j** The prognostic significance of IL6 was confirmed in REMBRANDT gliomas. ***P < 0.001. Classical, CL; neural, NE; proneural, PN; mesenchymal, ME

We first examined IL6 and IL6R mRNA expression in clinical gliomas using TCGA dataset. We found that the levels of IL6 and IL6R expression were significantly higher in mesenchymal subtypes of glioma compared with classical, neural, and proneural subtypes, respectively (Fig. 1a–b). Moreover, compared with the IDH-mutant gliomas, the levels of IL6 and IL6R mRNA expression were higher in IDH-wildtype gliomas (Fig. 1c–d). In addition, upregulation of both IL6 and IL6R was associated with decreased survival rates in both TCGA and REMBRANDT gliomas (Fig. 1e–j).

**Fig. 2:**
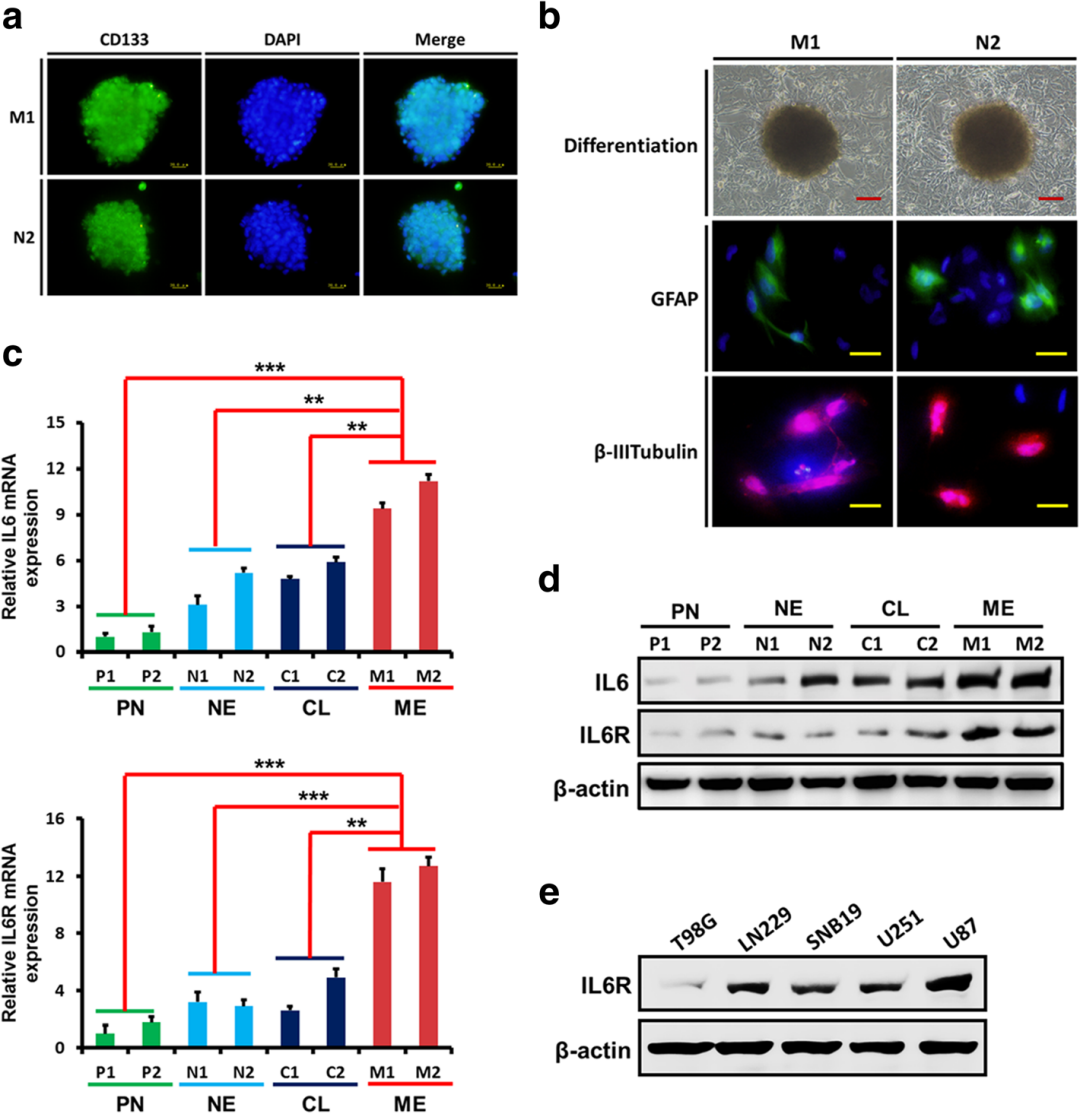
The expression of IL6 and IL6R in patient-derived primary glioma cells. **a–b** Neurospheres comprised of CD133-positive glioma stem-like cells (GSCs) obtained from primary cultures (**a**) became adherent and differentiated into GFAP- or β III tubulin -positive cells (**b**). Scale bars: red = 50 μm, yellow = 25 μm. **c**-**d** qPCR (**c**) and western blotting analyses (**d**) showed that, compared with the classical, neural, and proneural glioma cells, the levels of IL6 and IL6R mRNA and protein expression were higher in mesenchymal glioma cells. **e** The expression of IL6R in different glioma cell lines was examined by western blotting. **P < 0.01, ***P < 0.001. Classical, CL; neural, NE; proneural, PN; mesenchymal, ME

Using freshly isolated clinical glioma specimens, we performed primary cell culture and GSCs isolation (Fig. 2a–b) [18]. Next, we examined the expression of IL6 and IL6R in patient-derived primary glioma cells. Compared with the classical, neural, and proneural glioma cells, the levels of IL6 and IL6R mRNA and protein expression were higher in mesenchymal glioma cells (Fig. 2c–d). Together, these findings reveal that elevated IL6 signalling is associated with more aggressive subtypes of gliomas and contributes to poor patient outcome.

### IL6R knockdown inhibits glioma cell growth and invasion

**Fig. 3:**
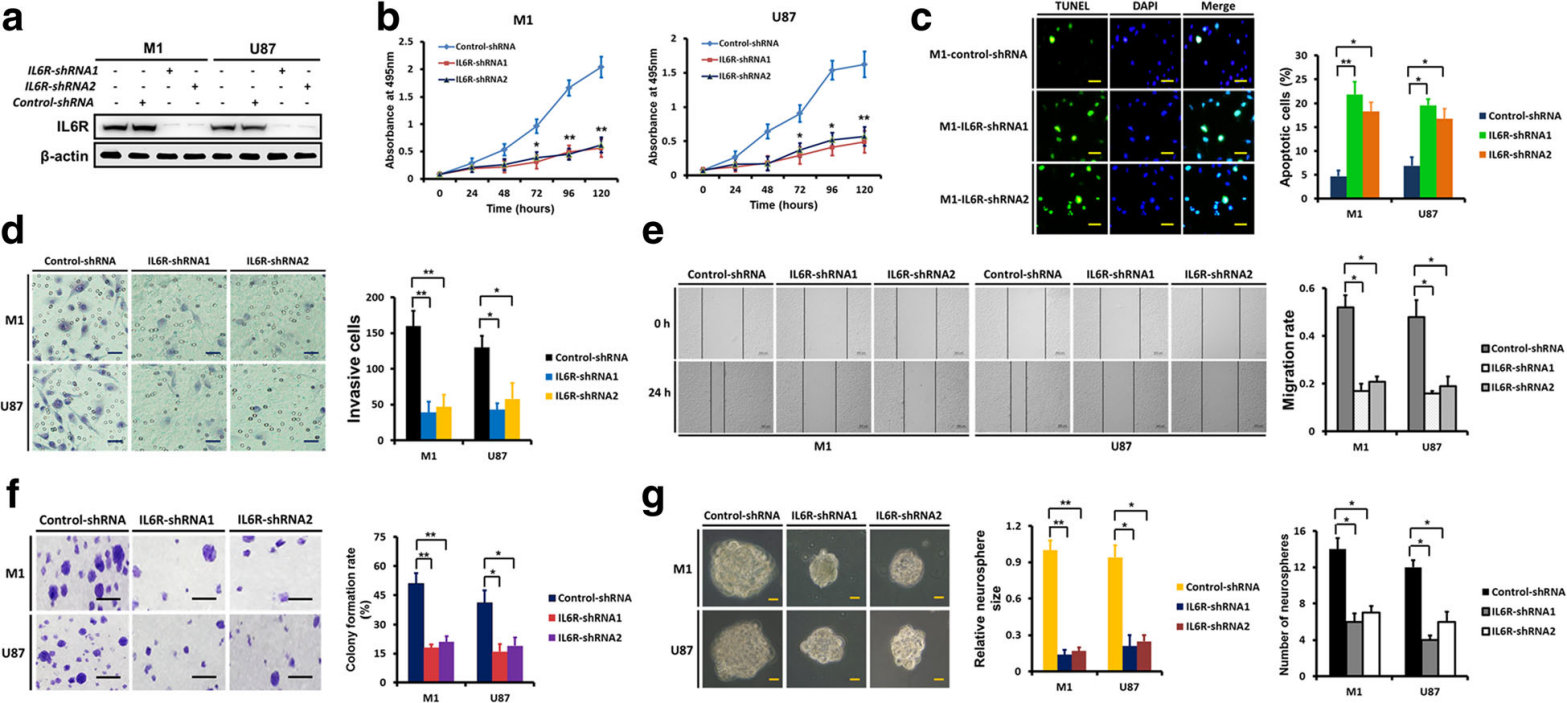
IL6R knockdown inhibits the growth and invasion of glioma cells. **a** IL6R protein levels in M1 and U87 cells that were transfected with IL6R-shRNA1, IL6R-shRNA2 or control-shRNA, respectively, were tested by western blot analyses, and IL6R knockdown was confirmed. **b** Targeting of IL6R via specific shRNAs significantly decreased the proliferation of M1 and U87 cells as assessed with MTS assay. **c** Representative microphotographs showing the detection of apoptosis using the TUNEL assay in control and IL6R silenced M1 cells. Scale bar = 25 μm. Histogram showing the quantification of the apoptotic rate. **d** Representative microphotographs showing the invasion of M1 and U87 cells in the presence of IL6R-shRNA or control-shRNA using the matrigel assay. Scale bar = 50 μm. Histogram showing the quantification of invasive cells. **e** The migration of M1 and U87 cells in the presence of IL6R-shRNA or control-shRNA was tested using the wound healing assay. Scale bar = 500 μm. Histogram showing the quantification of the migration rate. **f** IL6R knockdown decreased the colony formation rate of M1 and U87 cells. Scale bar = 50 μm. **g** Representative images of the effect of IL6R knockdown on M1 and U87 GSC neurospheres. Scale bars = 50 μm. Histograms showing the quantification of relative sizes and numbers of indicated neurospheres. Results are presented as mean ± SEM of triplicate samples from three independent experiments. *P < 0.05 and **P < 0.01

To evaluate the functional significance of IL6R upregulation in gliomas, we knocked-down the expression of IL6R in M1 cells and U87 cells, both of which normally have high IL6R expression levels, (Fig. 2c–e) using two specific shRNAs (Fig. 3a). IL6R silencing dramatically reduced cell growth associated with decreased proliferation over time (Fig. 3b) and increased cell apoptosis (2–3-fold; Fig. 3c). IL6R knockdown also inhibited glioma cell invasion 2.2–3.1-fold (Fig. 3d) and migration 1.5–2.4-fold (Fig. 3e). As shown by soft-agar colony formation, loss of IL6R expression significantly decreased glioma cells tumorigenesis in vitro 1.5–1.8-fold (Fig. 3f). Moreover, depletion of IL6R in M1 and U87 GSCs resulted in marked reductions in neurosphere size (2.7–6.1-fold) and number (1.3–1.5-fold; Fig. 3g). These data demonstrate that IL6R mediated signals are important for glioma growth and invasion.

### IL6R overexpression promotes glioma cell growth and invasion

**Fig. 4:**
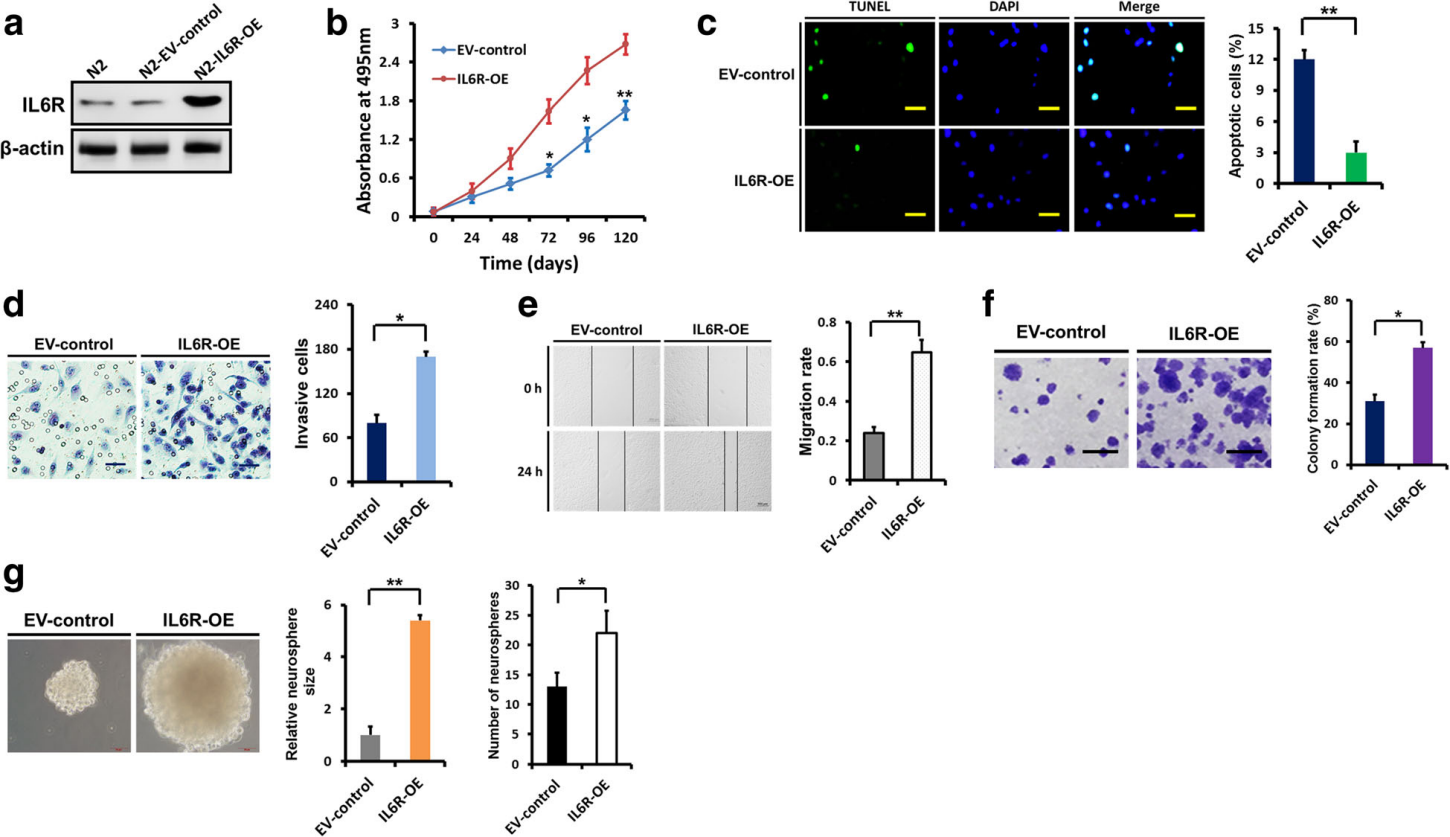
IL6R overexpression enhances the growth and invasion of glioma cells. **a** In N2 cells, IL6R was overexpressed several-fold compared with the empty vector transfected N2 cells with low endogenous IL6R expression. **b** IL6R overexpression markedly increased the proliferation of N2 cells, as evaluated by the MTS assay. **c** Representative microphotographs showing the detection of apoptotic cells using the TUNEL assay in control and IL6R overexpressing N2 cells. Scale bar = 25 μm. Histogram showing the quantification of the apoptotic rate. **d** Representative microphotographs showing that overexpression of IL6R in N2 cells resulted in increased invasion through matrigel-coated filters. Scale bar = 50 μm. Histogram showing the quantification of the invasive cells. **e** The wound healing assay showing that overexpression of IL6R in N2 cells promoted migration. Scale bar = 500 μm. Histogram showing the quantification of the migration rate. **f** IL6R overexpression increased the colony formation rate of N2 cells. Scale bar = 50 μm. **g** Representative images of the effect of IL6R overexpression on N2 GSC neurospheres. Scale bars = 50 μm. Histograms showing the quantification of relative sizes and numbers of indicated neurospheres. Results are presented as mean ± SEM of triplicate samples from three independent experiments. *P < 0.05 and **P < 0.01. OE: overexpression; EV: empty vector

To further confirm the role of IL6R in glioma growth and invasion, we overexpressed IL6R in N2 cells with low levels IL6R (Figs. 2c–d and 4a). We observed a significant increase in cell proliferation (Fig. 4b) and corresponding decrease in cell apoptosis (more than 3-fold; Fig. 4c) after IL6R overexpression. IL6R overexpression enhanced N2 cells invasion 1.5-fold (Fig. 4d) and migration 1.7-fold (Fig. 4e). A marked increase in colony formation rate was observed after IL6R overexpression (Fig. 4f). In addition, overexpression of IL6R in N2 GSCs resulted in significant increases in neurosphere size (4.4-fold) and number (1.7-fold; Fig. 4g).

### IL6R expression affects glioma cells tumorigenesis in vivo

**Fig. 5:**
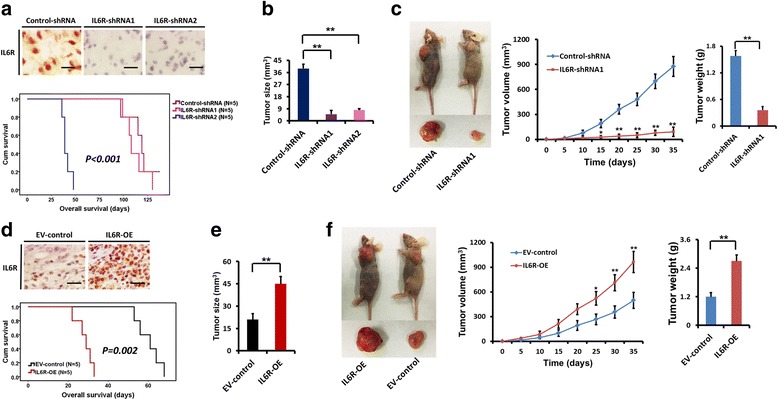
Role of IL6R in tumour growth in vivo. **a** IHC staining for IL6R from intracranial tumours demonstrating IL6R silencing in M1-IL6R-shRNA1 and M1-IL6R-shRNA2 injected mice. Scale bar = 25 μm. Kaplan–Meier survival curves show the survival differences of mice injected with M1-control-shRNA, M1-IL6R-shRNA1 and M1-IL6R-shRNA2 cells, respectively. **b** IL6R knockdown significantly inhibited intracranial tumour growth. **c** IL6R silencing significantly inhibited subcutaneous tumour growth. **d** IHC staining for IL6R from intracranial tumours demonstrating IL6R overexpression in N2-IL6R-OE injected mice. Scale bar = 25 μm. Kaplan–Meier survival curves show the survival differences of mice injected with N2-EV-control and N2-IL6R-OE cells, respectively. **e** IL6R overexpression promoted intracranial tumour growth. **f** IL6R overexpression significantly increased subcutaneous tumour growth. *P < 0.05 and **P < 0.01

To determine the role of IL6R in glioma tumourigenesis in vivo, we separately implanted M1-control-shRNA, M1-IL6R-shRNA1, M1-IL6R-shRNA2, N2-EV-control or N2-IL6R-OE cells into the brains of mice. IHC staining for IL6R from in vivo tumours demonstrated either IL6R silencing or overexpression (Fig. 5a and d). Compared with M1-control-shRNA, knockdown of IL6R significantly suppressed intracranial tumour growth and resulted in an increase in survival (median survival: 39 ± 1.6 days vs. 119 ± 4.4 and 108 ± 2.2 days, respectively; Fig. 5a–b). In addition, IL6R overexpression significantly promoted intracranial tumour growth and decreased survival of tumour-bearing mice (median survival: 61 ± 5.4 days vs. 29 ± 2.1 days, respectively; Fig. 5d–e). In subcutaneous xenograft models, IL6R knockdown resulted in obvious growth delay of M1 cells, while IL6R overexpression led to significantly increased tumour growth of N2 cells (Fig. 5c and f).

### IL6R is important for IL6-stimulated glioma cell growth and invasion

**Fig. 6:**
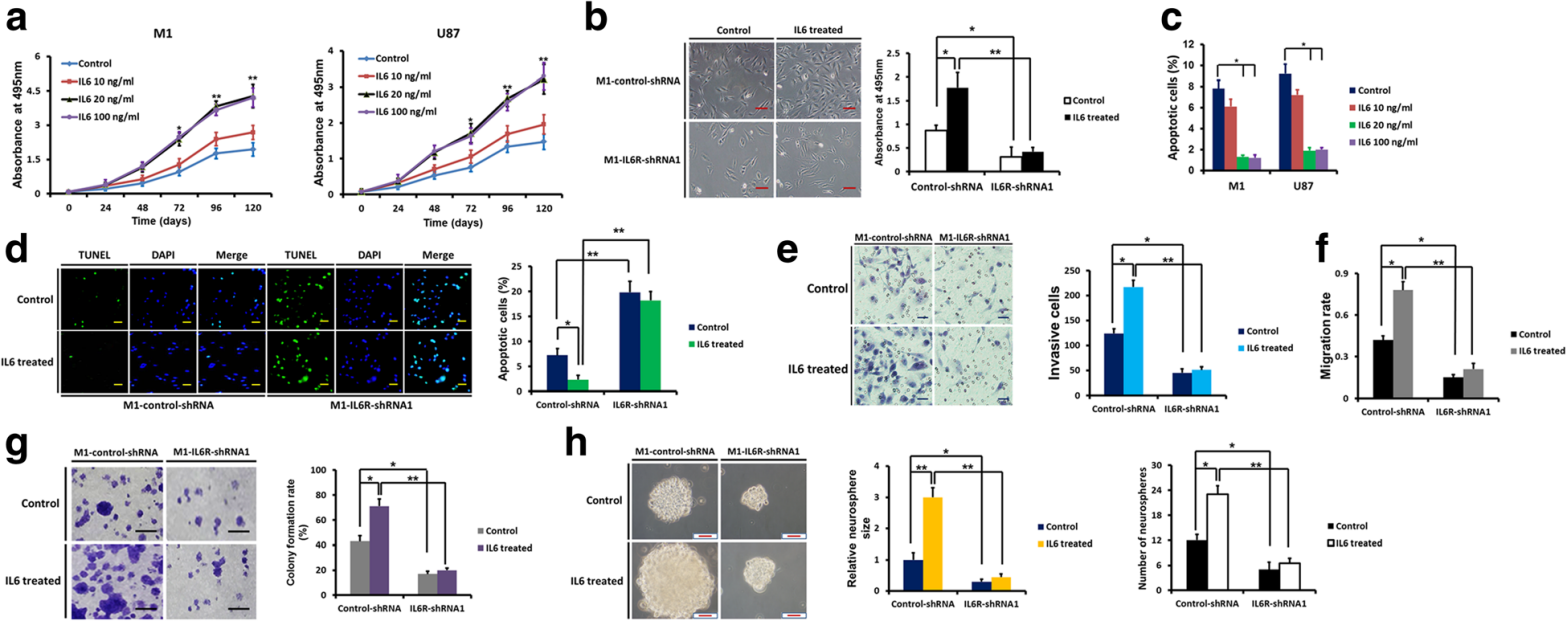
IL6R is required for IL6-stimulation induced glioma cell growth and invasion. **a** The effect of IL6-stimulation at different concentrations on the proliferation of M1 and U87 cells, as assessed by the MTS assay. **b** MTS assay results showed that silencing of IL6R remarkably decreased IL6-stimulation induced proliferation of M1 cells. Scale bar = 100 μm. **c** The effect of IL6-stimulation at different concentrations on apoptosis of M1 and U87 cells, as examined by the TUNEL assay. **d** TUNEL assay demonstrated that IL6R knockdown markedly abrogated the anti-apoptotic effect of IL6 in M1 cells. Scale bar = 50 μm. **e** Knockdown of IL6R reversed the IL6-induced invasive potential of M1 cells, as assessed by the matrigel assay. Scale bar = 50 μm. **f** Wound healing assay demonstrated that silencing of IL6R significantly inhibited IL6-induced migration of M1 cells. **g** IL6R knockdown significantly decreased the colony formation ability of M1 cells treated with IL6 treatment. Scale bar = 50 μm. **h** In M1 GSCs, IL6-stimulation increased neurosphere size and number, which was also mainly dependent on the expression of IL6R. Scale bar = 50 μm. *P < 0.05 and **P < 0.01

Firstly, we examined the effect of IL6 stimulation on glioma cell proliferation and apoptosis by treating M1 and U87 cells with different concentrations of IL6. We found that IL6-stimulation induced proliferation (Fig. 6a-b) and inhibited apoptosis (Fig. 6c) at 20 ng/ml. However, the effects of IL6 treatment were largely dependent on the expression of IL6R (Fig. 6b and d). Moreover, IL6-stimulation markedly increased M1 cell invasion, migration and colony formation, while IL6R silencing obviously abrogated the effects of IL6-stimulation (Fig. 6e–g). In M1 GSCs, IL6-stimulation dramatically increased neurosphere size and number, which was also mainly dependent on the expression of IL6R (Fig. 6h).

### The expression of IL6R and IL6 is regulated by NFAT1 in glioma cells

**Fig. 7:**
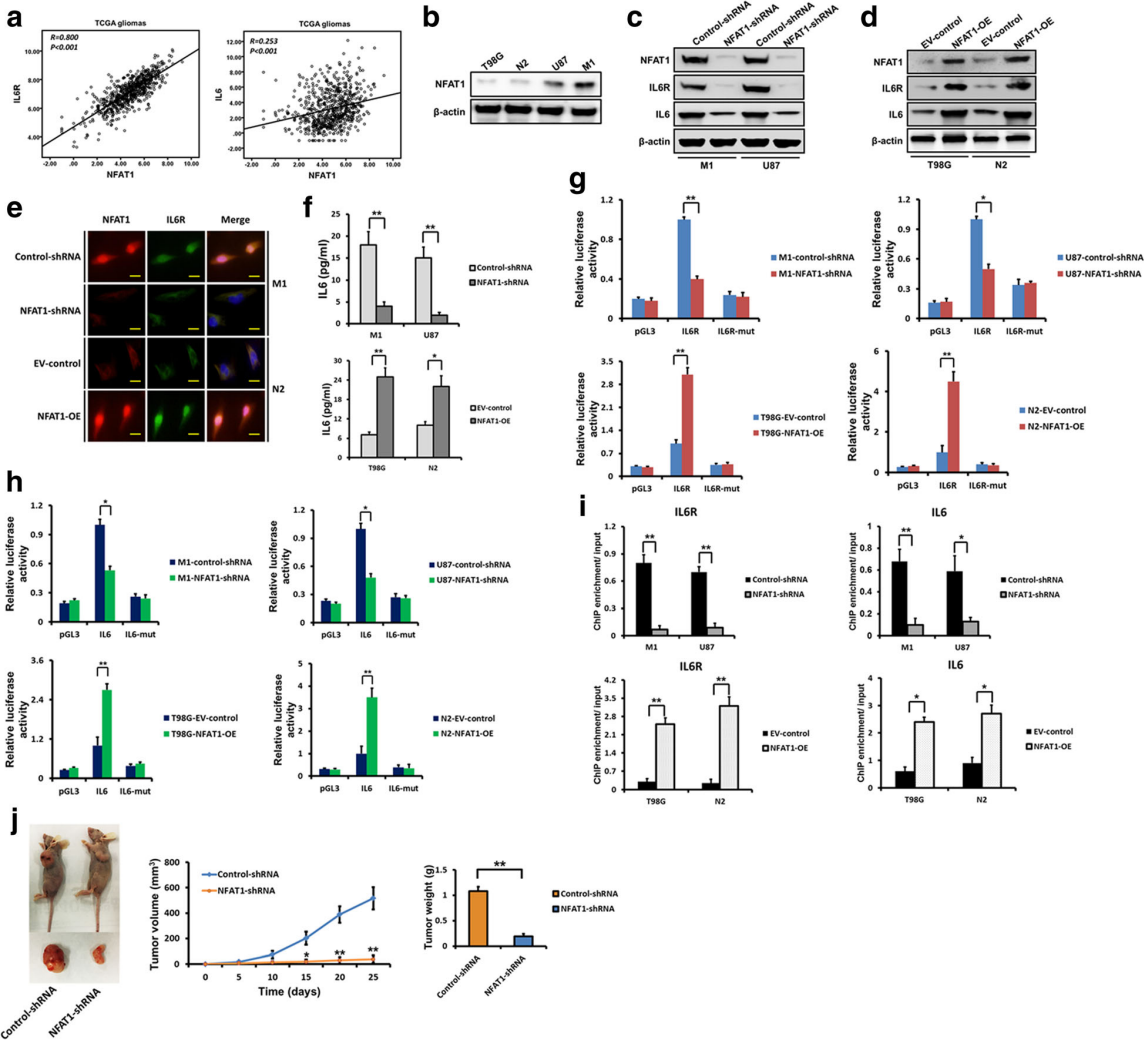
The expression of IL6R and IL6 is regulated by NFAT1 in glioma cells. **a** Expression data of IL6R, IL6 and NFAT1 mRNA in gliomas were downloaded from TCGA dataset and analysed. The expression of IL6R and IL6 were significantly correlated with that of NFAT1. **b** The expression of NFAT1 in different glioma cells was examined using western blotting. **c** Effect of NFAT1 knockdown on the expression of IL6R and IL6 was examined by western blotting in M1 and U87 cells. **d** Effect of NFAT1 overexpression on the expression of IL6R and IL6 was examined by western blotting in T98G and N2 cells. **e** Double-labelled immunofluorescence staining showing that the downregulation of NFAT1 is accompanied decreased IL6R expression in M1 cells, while upregulation of NFAT1 is accompanied increased IL6R expression in N2 cells. Scale bar =10 μm. **f** The levels of IL-6 secretion were examined by ELISA in cell-free supernatants from glioma cells after NFAT1 silencing or overexpression. **g-h** Effect of NAFT1 on IL6R (**g**) and IL6 (**h**) promoter activities. Silencing NFAT1 in both M1 and U87 cells significantly reduced the luciferase activity driven by the wildtype IL6R and IL6 promoters compared with control-shRNA. Mutating NFAT1 binding sites resulted in reduced promoter activity compared with the wildtype promoter. NFAT1 overexpression in T98G and N2 cells increased luciferase promoter activities. **i** Binding of NFAT1 to IL6R and IL6 promoters. Binding of NFAT1 was decreased when NFAT1 was silenced in M1 and U87 cells, while binding was increased when NFAT1 was overexpressed in T98G and N2 cells. **j** In M1 cells, NFAT1 knockdown significantly inhibited subcutaneous tumour growth. *P < 0.05 and **P < 0.01

Next, we sought to identify the regulatory factors involved in IL6 signalling in glioma cells. According to TCGA dataset, the expression of IL6R was significantly correlated with the expression of NFAT1 (*R* = 0.800, *P* < 0.001; Fig. 7a), as was the expression of IL6 (*R* = 0.253, *P* < 0.001; Fig. 7a). To determine the regulatory effect of NFAT1 on the expression of IL6R and IL6, we knocked-down NFAT1 in M1 and U87 cells that highly express NFAF1, and also overexpressed NFAT1 in N2 and T98G cells, which have low endogenous levels of NFAT1 (Fig. 7b). As shown in Fig. 7c–f, western blotting, immunofluorescence and ELISA demonstrated that NFAT1 knockdown resulted in decreased expression of IL6R and reduced expression and secretion of IL6, while NFAT1 overexpression augmented the expression of IL6R and increased expression and secretion of IL6.

To determine whether NFAT1 regulates IL6R and IL6 at the transcriptional level, luciferase reporter assays were designed so that the IL6R and IL6 promoters were cloned upstream of the luciferase. The promoter was designed with and without mutations in the IL6R and IL6 promoter NFAT1-binding site. NFAT1 knockdown significantly reduced luciferase activity in M1 and U87 cells with wild-type promoters, while overexpression of NFAT1 in N2 and T98G cells significantly increased IL6R and IL6 promoter activity (Fig. 7g–h). Promoter mutation also markedly decreased luciferase activity in comparison with the wild-type promoter (Fig. 7g–h). ChIP assays were then performed to confirm the binding of NFAT1 to the IL6R and IL6 promoters. NFAT1 bound to the promoters of IL6R and IL6 in both M1 and U87 cells, and following NFAT1 knock-down, binding was significantly inhibited (Fig. 7i). Also, following NFAT1 overexpression in N2 and T98G cells, binding of NFAT1 to the respective binding sites were markedly increased (Fig. 7i). Therefore, these results show that NFAT1 enhances the promoter activities of IL6R and IL6 in glioma cells. Moreover, in subcutaneous xenograft models, NFAT1 knockdown resulted in remarkable growth delay of M1 cells (Fig. 7j).

## Discussion

Immuno-inflammatory factors play an important role in the tumourigenesis and malignant progression of gliomas. Although the inflammatory milieu may precede glioma development, glioma can later harness the expression of inflammatory mediators and establish new immuno-microenvironments to facilitate proliferation and migration [26]. Gliomas have been shown to initiate the downregulation of immune-related protective genes and upregulate oncogenic inflammatory factors, therefore gaining a survival advantage [4]. Previous studies determined that IL6 signalling in gliomas acted as a key regulator of the immunosuppressive microenvironment and was an important promoter of proliferation, survival and invasiveness of tumour cells [27–30].

In the present study, using TCGA database and patient-derived primary glioma cells, we found both IL6 and IL6R expression were significantly correlated with mesenchymal subtype and IDH-wildtype gliomas, and were predictors for unfavourable prognosis. Consistent with our results, mesenchymal gliomas have been associated with a higher local inflammatory reaction and more aggressive behaviour [19], and therefore, IL6 signalling may be a major player maintaining the malignant phenotype of mesenchymal gliomas [29, 30]. The strong association between IDH-wildtype gliomas and upregulation of IL6 and IL6R expression suggests that the IL6 signalling may contribute to the poor prognosis of patients with wild-type IDH1, although the exact mechanism requires further research.

IL6 may function as an autocrine and/or paracrine factor, and the high levels of IL6 in the glioma microenvironment could be the result of secretion by immune-infiltrating cells and stromal cells, in addition to tumour cells [6]. A previous study demonstrated that as a stromal component of gliomas, mesenchymal stem cells could secrete IL6, which increased proliferation and maintained the stemness of GSCs [31]. These results indicated that, due to the wide range of IL6 sources, it is difficult to deplete IL6 in the inflammatory milieu of gliomas. Importantly, we found that IL6-stimulated glioma growth and invasion was largely dependent on the expression of IL6R. IL6R knockdown significantly inhibited the tumourigenesis of glioma cells both in vitro and in vivo. Therefore, IL6R of the tumour cells might be a better target than IL6 for the potential novel treatment of gliomas.

NFATs are essential regulatory transcriptional factors of the immune system and control the expression of numerous cytokines and their receptors [32]. Recent studies suggest that NFATs may be involved in many aspects of cancer, including carcinogenesis, cancer cell survival, invasion and the tumour microenvironment [33]. We previously demonstrated that the expression of NFAT1 is upregulated in GBMs compared with low-grade gliomas, suggesting a role of NFAT1 in the malignant progression of gliomas. Moreover, NFAT1 regulates the invasion of GBM cells [34]. In the present study, we showed that NFAT1 regulates the expression of IL6 and IL6R at the transcriptional level. These findings suggest that IL6 and IL6R may be the downstream targets of NFAT1 pathway. In support of this theory, a previous study showed that NFAT1 activation increased the expression of IL6 and promoted the pathogenesis in giant cell arteritis [35]. Another study reported the induction of IL6 by NF-κB [36], however, as immune-related transcriptional factors, NFAT1 and NF-κB often cooperate with each other in the regulation of cytokine expression [37, 38]. In gliomas, the NFAT1/IL6/IL6R pathway may mediate crosstalk between tumours and immune cells resulting in a tumour-promoting inflammatory microenvironment.

## Conclusion

IL6 signalling mediated by NFAT1 contributes to aggressive phenotypes of gliomas, which suggests an important role of immunomodulatory factors in glioma malignant progression.

## Additional files

Additional file 1: Figure S1. The mRNA expression markers and molecular classification of patient-derived primary glioma cells. A-F: Real time-PCR was performed to detect the expression of the proneural (PN) marker PDGFRA and OLIG2, neural (NE) marker NEFL, classical (CL) marker EGFR, mesenchymal (ME) marker FN1 and YKL40. And patient-derived primary glioma cells were classified into the four molecular subtypes. The PCR primers are shown in Additional file 2: Table S1. (TIFF 1483 kb)

Additional file 2: Table S1. PCR Primers. (DOC 35 kb)

## Abbreviations

CGGA: Chinese Glioma Genome Atlas
ChIP: Chromatin immunoprecipitation
ELISA: Enzyme-linked immunosorbent assay
GBM: Glioblastoma multiforme
GFAP: Glial fibrillary acidic protein
GSC: Glioma stem-like cell
IDH: Isocitrate dehydrogenase
IHC: Immunohistochemistry
IL6: Interleukin-6
IL6R: Interleukin-6 receptor
NFAT: Nuclear factor of activated T cells
TCGA: The Cancer Genome Atlas
TUNEL: Terminal dexynucleotidyl transferase(TdT)-mediated dUTP nick end labeling
