# Supplementary figures and images for: NFAT1-regulated IL6 signalling contributes to aggressive phenotypes of glioma
Source: Cell Commun Signal. 2017 Dec 19;15:54. doi: 10.1186/s12964-017-0210-1 (PMC5735798; doi:10.1186/s12964-017-0210-1)

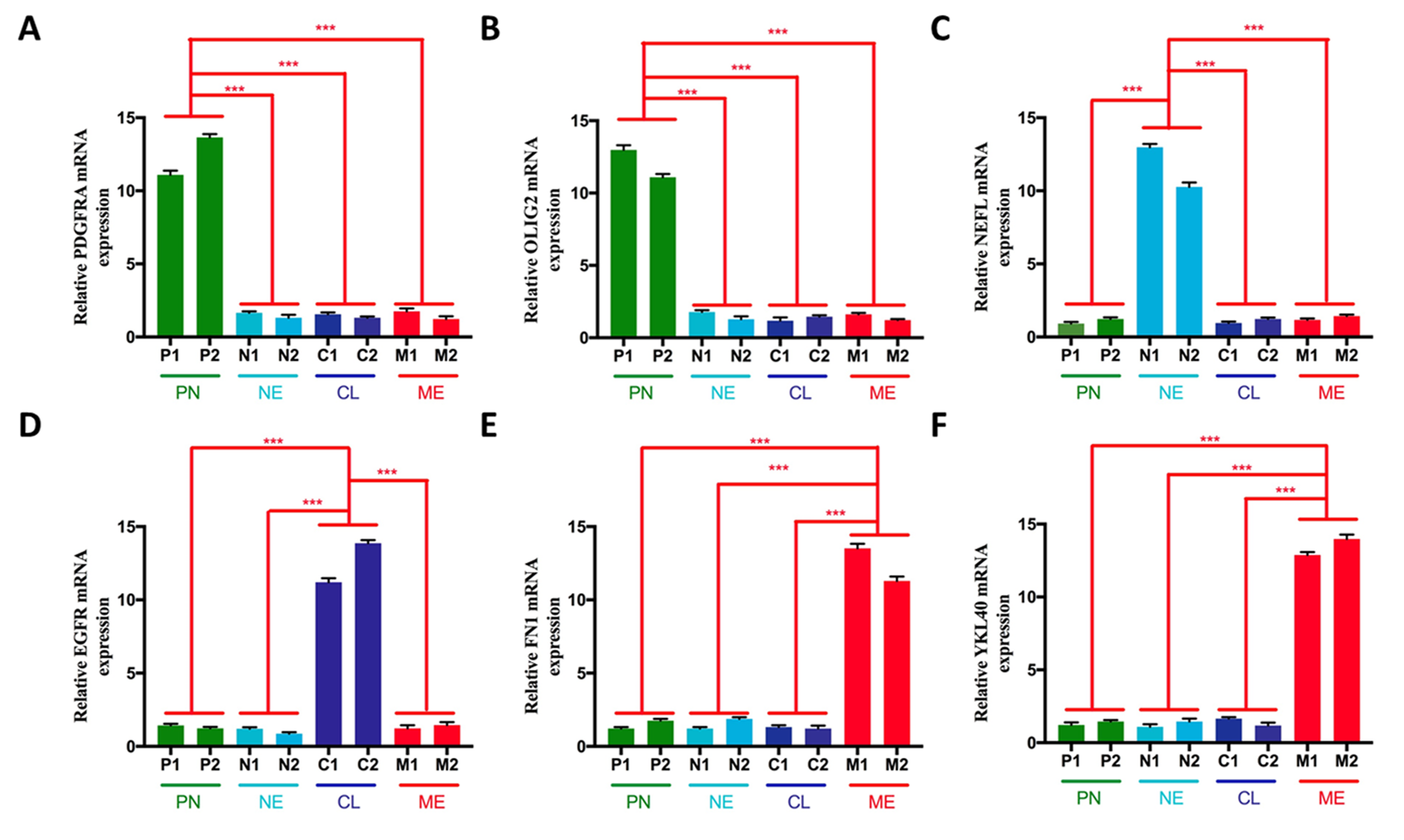

Supplement: Supplementary file 1 — The mRNA expression markers and molecular classification of patient-derived primary glioma cells. A-F: Real time-PCR was performed to detect the expression of the proneural (PN) marker PDGFRA and OLIG2, neural (NE) marker NEFL, classical (CL) marker EGFR, mesenchymal (ME) marker FN1 and YKL40. And patient-derived primary glioma cells were classified into the four molecular subtypes. The PCR primers are shown in Additional file 2: Table S1. (TIFF 1483 kb) [file 12964_2017_210_MOESM1_ESM.tif]
