# Supplementary material for: NFAT1-regulated IL6 signalling contributes to aggressive phenotypes of glioma
Source: Cell Commun Signal. 2017 Dec 19;15:54. doi: 10.1186/s12964-017-0210-1 (PMC5735798; doi:10.1186/s12964-017-0210-1)
Supplement: Supplementary file 2 — PCR Primers. (DOC 35 kb) [file 12964_2017_210_MOESM2_ESM.doc]

**Table S1. PCR Primers**

| **Primer** | **Forward (5’-3’)** | **Reverse (5’-3’)** |
| --- | --- | --- |
| PDGFRA | TGGCAGTACCCCATGTCTGAA | CCAAGACCGTCACAAAAAGGC |
| OLIG2 | CCAGAGCCCGATGACCTTTTT | CACTGCCTCCTAGCTTGTCC |
| NEFL | ATGAGTTCCTTCAGCTACGAGC | CTGGGCATCAACGATCCAGA |
| EGFR | AGGCACGAGTAACAAGCTCAC | ATGAGGACATAACCAGCCACC |
| FN1 | CGGTGGCTGTCAGTCAAAG | AAACCTCGGCTTCCTCCATAA |
| YKL40 | GTGAAGGCGTCTCAAACAGG | GAAGCGGTCAAGGGCATCT |
| IL6 | ACTCACCTCTTCAGAACGAATTG | CCATCTTTGGAAGGTTCAGGTTG |
| IL6R | CCCCTCAGCAATGTTGTTTGT | CTCCGGGACTGCTAACTGG |
| NFAT1 | GAGCCGAATGCACATAAGGTC | CCAGAGAGACTAGCAAGGGG |
| β-actin | CATGTACGTTGCTATCCAGGC | CTCCTTAATGTCACGCACGAT |
